# Editorial: Microbial diversity and ecosystem functioning in wetlands

**DOI:** 10.3389/fmicb.2024.1414288

**Published:** 2024-05-06

**Authors:** Jing Li, Huajing Li, Meng Li, Wenbing Li, Juntao Wang

**Affiliations:** ^1^Institute of Wetland Research, Chinese Academy of Forestry, Beijing Key Laboratory of Wetland Ecological Function and Restoration, Beijing, China; ^2^Beijing Hanshiqiao National Wetland Ecosystem Research Station, Beijing, China; ^3^Institute of Ecological Conservation and Restoration, Chinese Academy of Forestry, Beijing, China; ^4^School of Life and Environmental Sciences, Hangzhou Normal University, Hangzhou, China; ^5^Hawkesbury Institute for the Environment, Western Sydney University, Penrith, NSW, Australia; ^6^Global Centre for Land-Based Innovation, Western Sydney University, Penrith, NSW, Australia; ^7^School of Science, Western Sydney University, Penrith, NSW, Australia

**Keywords:** wetlands, microbial diversity, ecosystem function, relationship, drivers

Microbes are key drivers on wetland ecological processes, such as nutrient cycling, pollutants degradation, and greenhouse gas emission. Despite the increasing studies of microbial community in driving wetland ecosystem processes, the mechanism of the relationship between microbial communities and key ecosystem functions remains poorly understood; in addition, the relationship between microbial diversity and wetland functions in different types of wetlands has not been analyzed in sufficient details. The diversity of microbial communities can be affected by abiotic (including biological matter, hydrological conditions, and temperature) and biological factors (such as typical plants). Understanding the relationships between microbial diversity and wetland functions in different types of wetlands is crucial for studying wetland ecological processes.

The aim of this Research Topic is to clarify the characteristics and functions of microorganisms in different types of wetlands, and underlying mechanisms of the relationships between microbial communities and ecological functions. Within this topic, 6 articles have been published, fostering new ideas for the exploration of complex wetland ecosystems through microbial research.

After accounting for the host selection effect of halophyte, Wang et al. investigated the distribution of bacterial communities in the rhizosphere (30–40 cm) of typical coastal halophyte species (*Phragmites australis* and *Suaeda salsa*) in temperate and subtropical salt marshes, spanning 1,100 km in eastern China. It's a study on a broad spatial scale. Their research revealed that the α-diversity of bacterial communities in the sediment of temperate salt marsh was higher than that of subtropical salt marsh. The stronger connections and more complex network structures were observed in the temperate salt marsh, while more cohesive modular structure of bacterial network was shown in subtropical salt marsh. Soil properties, geographic factors, and plant functional traits collectively shape bacterial communities in salt marsh, and soil chemical properties as well as root exudates regarded as most important predictors.

To unravel the dynamics of water and sediment bacteria under different plant covers, in the Yellow River floodplain, Han et al. conducted an investigation about changes of bacterial diversity and community structure in sediment (at depths of 0–10 cm and 10–20 cm) as well as water under the influence of different plant species (*Phragmites australis (Cav.) Trin. ex Steud*. and *Erigeron canadensis L*.). Their study revealed that there was a limited overlap of interactions between the bacterial community of water and sediment. Interestingly, the different plant cover and sediment depths significantly influenced the structure of the bacterial community in the sediment rather than that in the water.

Chen et al. studied the relationship between seasonal changes of bacterial communities and environmental factors in inland freshwater lakes. They compared the diversity of soil bacteria in the surface soil (5–15 cm) of Nansi Lake Wetland in summer and winter. Specifically, they found a higher microbial diversity during the summer. Temperature and the available phosphorus may be the key factors influencing the seasonal variation of bacterial diversity.

Most of research concerning the impact of salinity on methanotrophic communities have been limited to controlled experiments conducted in laboratory. Zhang et al. studied the broader regional-scale effects of salinity on the diversity and composition of methanotrophic communities in natural lake sediments. They filled the knowledge gap by investigating sediment samples collected from 13 lakes in Inner Mongolia, China. Their study showed that difference in the community structure of methanotrophs between the hypersaline sediment samples (salinity >0.69%) and the hyposaline samples (salinity < 0.69%). Notably, salinity was significantly negatively correlated with methanotrophic diversity, indicating the higher of the salinity with the lower diversity of methanotrophic community, and the methanotroph network was more closely connected at low salinity.

Functional diversity is a link between biodiversity and ecosystem functioning. Barros et al. conducted a study of microbial functional diversity based on the physiological profile at the community level. They focused on the functional diversity of soil communities (0–15 cm) in Amazonian floodplains flooded with different water types: black water (Negro River), clear water (Tocantins River), and white water (Solimões River). There are significant differences in the metabolic activity of soils under different Amazonian water types, and the general trend was clear water floodplain > black water floodplain > white water floodplain. It's important to consider soils under the influence of flood pulses, water types, and land use as environmental factors when recognizing functional diversity of microbial communities and ecosystem functioning in Amazonian floodplains.

Climate warming has the potential to induce drying of Arctic wetlands. To unravel the response of soil micro-eukaryotes to increasing drying in Arctic wetlands, Myeong et al. investigate soil micro-eukaryotic communities in the surface layer (0–7.5 cm) and subsurface layer (7.5–15 cm) of the Siberian wet tundra under a decade-long drainage manipulation. They found that the abundance of micro-eukaryotic communities increased under the drainage. Moreover, the study showed that fungal communities was more strongly influenced by drainage-induced vegetation change than drainage treatment itself, whereas the vegetation effect on non-fungal micro-eukaryotic communities was relatively weak. Integrating micro-eukaryotes into the belowground microbiome framework will enhance our understanding of the functioning of belowground ecosystems in the Arctic.

In summary, although this Research Topic helps us to better understand the effects of particular environmental factor on microorganisms, few studies conduct long-term monitoring, which might lead to limited understanding of wetland microbes on temporal scales. What's more, studies are still needed on the coupling between the shifts of wetland microbial communities with the changes of ecosystem functions, understanding how the succession of microbial community could drive wetland ecosystem functions.

## Author contributions

JL: Writing – original draft, Writing – review & editing. HL: Writing – original draft. ML: Supervision, Writing – review & editing. WL: Supervision, Writing – review & editing. JW: Supervision, Writing – review & editing.

